# A novel electromagnetic apparatus for *in-situ* synchrotron X-ray imaging study of the separation of phases in metal solidification

**DOI:** 10.1016/j.ohx.2020.e00104

**Published:** 2020-03-14

**Authors:** Billy Koe, Colin Abraham, Chris Bailey, Bob Greening, Martin Small, Thomas Connolley, Jiawei Mi

**Affiliations:** a Department of Engineering, University of Hull, Hull HU6 7RX, United Kingdom; b Diamond Light Source Ltd., Harwell Science and Innovation Campus, Didcot, Oxfordshire OX11 0DE, United Kingdom

**Keywords:** Electromagnetic phase separation, Synchrotron X-rays, In-situ solidification experiment, X-ray radiography and tomography

## Abstract

As a part of a research into new techniques for purifying recycled aluminium alloys, a novel electromagnetic apparatus had been developed for investigating in real-time the separation mechanisms of detrimental inclusions in aluminium alloy melts under alternating magnetic fields. The magnetic coil was designed based on the Helmholtz coil design. A viewing gap was designed for *in-situ* imaging studies using synchrotron X-rays. The gap was designed to maintain a uniform magnetic field in the central region where a sample is positioned. The current setup for the magnetic coil pair is able to produce a peak magnetic flux density of ~10 mT at a frequency of 25 kHz. A separate electrical resistance furnace, designed to concentrically fit within the magnetic coils, was used to control the heating (up to ~850°C) and cooling of the samples. After a series of systematic tests and commissioning, the apparatus was used in a number of *in-situ* and *ex-situ* experiments.

## Graphical abstract

**Figure:**
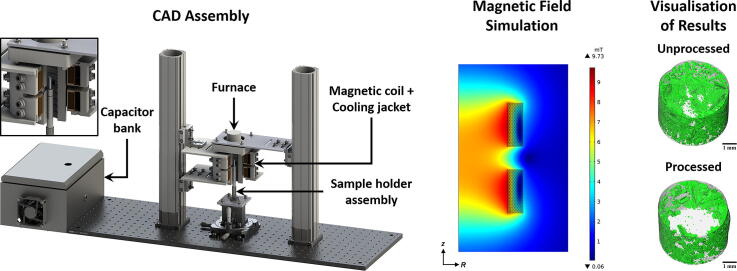

**Specifications table:** 

Hardware name	Electromagnetic Field Apparatus for Recycling Aluminium
Subject area	Engineering and Materials Science
Hardware type	Mechanical and electrical engineeringMaterials scienceAlternating magnetic field
Open Source License	Creative Commons Attribution 4.0 International
Cost of Hardware	~£3000.00 (Excluding general laboratory equipment)
Source File Repository	http://dx.doi.org/10.17632/ymr2yhrvyn.2

## 1 Context of the research

A recent report commissioned by the European Aluminium Association [1] predicted that the global demand for primary aluminium would reach ~107.8 million tonnes (Mt) by 2050, an increase of ~50% from the current level (~70 Mt). Currently, the annual global consumption of semi-fabricated aluminium products is approaching ~100 Mt, and it was forecasted to reach ~120 Mt by 2027 [1]. In Europe, the forecasted aluminium demand would be ~18 Mt by 2050 with approximately equal amount of primary and recycled aluminium in the market [1]. In recent years, much effort has been devoted to increasing the percentage of recycled aluminium in order to achieve a more sustainable aluminium supply in the coming age of circular economy. Using recycled aluminium to replace the primary aluminium would save up to 95% of energy in production and therefore significantly reduce the emission of greenhouse gases [1], [2] related to the production of primary aluminium.

Aluminium alloys used in high performance structural applications normally contain multiple alloying elements. Moreover, recycled aluminium alloys consist of a mixture of different alloy grades from the different sources which often contain other detrimental elements as well. For example, iron and silicon (apart from the Al-Si casting alloys) are the main elements that form irregular-shaped brittle phases that are detrimental to mechanical properties, especially ductility. Such detrimental phases prevent recycled aluminium from being used in high performance, high value structural applications such as the aerospace and the automotive industry. As a result, much of the recycled aluminium ends up in low-grade castings. Numerous processing techniques have been developed to deal with these impurities [3]. For example, the two most commonly used methods are separation via gravity and filtration using different medium. Separation under gravity relies on the density difference between the melt and the impurities [4]. Phases or elements of the similar density (e.g., aluminium and silicon) cannot be separated by the gravitational separation technique. In the case of filtration, the liquid metal is often continuously fed through filters of varying sizes to trap the inclusions within the melt [5], [6]. However, there is a need to frequently change new filters due to clogging of the filter pores. In addition, the limited filter pore size distribution is unable to trap inclusions of sizes outside the predefined size range.

Electromagnetic purification is a promising method for the production of “clean” metals [7]. Research on imposing various types of magnetic field for metal purification has been well documented in the literature. The application of an alternating magnetic field was confirmed as an efficient method in separating micrometre-sized inclusions [7]. In 1997, Yamao et al. [8] demonstrated the principle of separating alumina (Al_2_O_3_) inclusions from an aluminium melt by using a fixed alternating magnetic field with an 11-turn induction coil operating at frequencies of 3 and 33 kHz. A 7-turn induction coil capable of producing an alternating magnetic field with a frequency of 8.3 or 15.6 kHz was studied by Shu et al. [9] to separate the alumina inclusions from an aluminium alloy melt. Takahashi and Taniguchi [10], in 2003, used a 15-turn high frequency (30 kHz) induction furnace to investigate the separation of silicon carbide (SiC) particles within a pure aluminium melt. Guo et al. [11], [12] used a 9-turn induction coil, operating at a frequency of 20 kHz, for the separation of alumina inclusions and primary silicon crystals within a pure aluminium and an aluminium alloy melt, respectively.

## 2 Hardware description

**Fig. 1:**
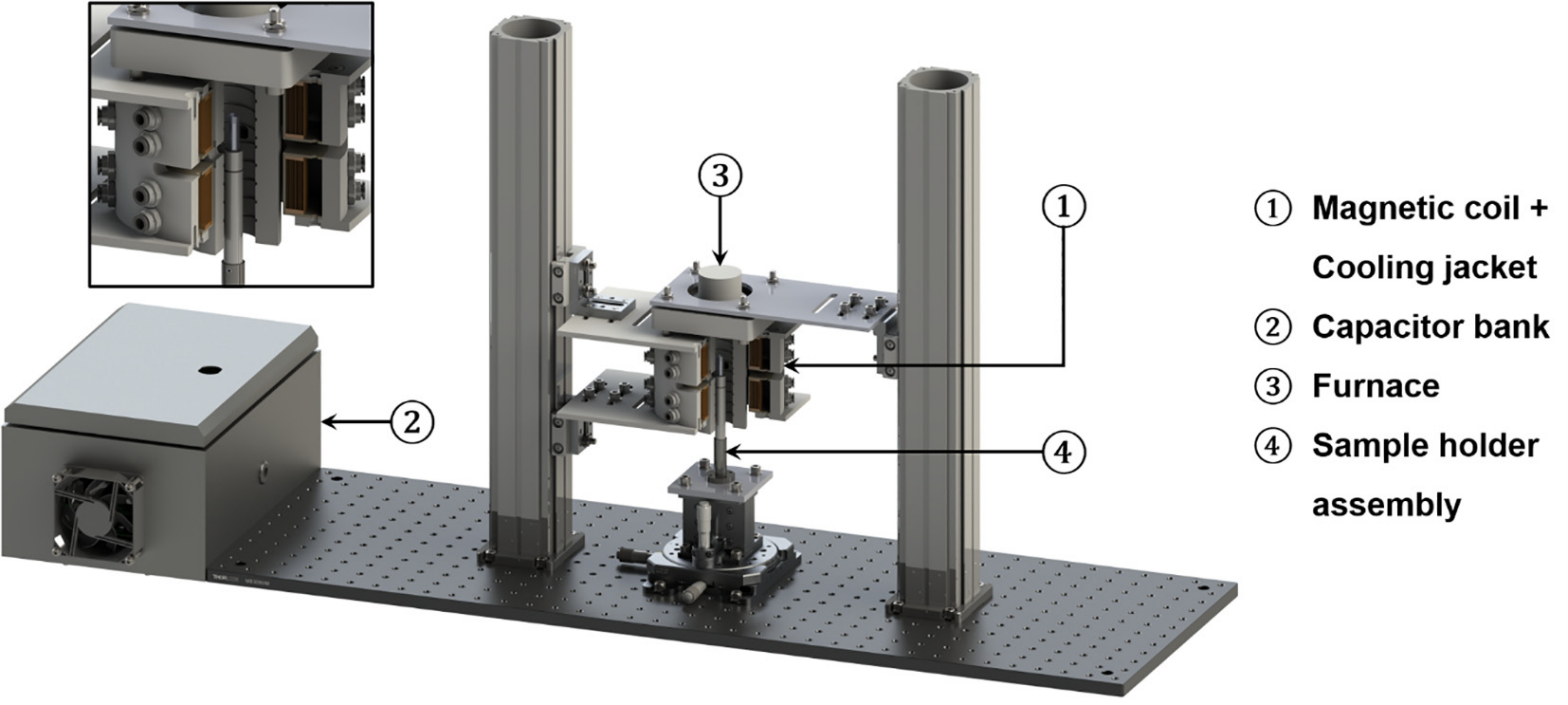
The 3D CAD assembly of the experimental apparatus (A quadrant of the magnetic coil, cooling jacket, and furnace was sectioned off). The inset shows the enlarged view of the sample positioned inside the furnace chamber.

The hardware consists of two stand-alone systems: a magnetic coil and a furnace. The 3D CAD assembly of the experimental apparatus is illustrated in Fig. 1. The motivation for designing and building such an apparatus was to investigate in real-time the motion of phases or inclusions (with different electrical conductivities) in molten alloy melts under an applied alternating magnetic field. Real-time data can provide unambiguous evidence for quantification of the separation efficiency of those phases in an alloy melt. The apparatus was designed for portability, for example, carrying out *ex-situ* experiments in a laboratory-based environment without X-ray beam, and *in-situ* synchrotron X-ray imaging experiments, for example, on Beamline I12 - JEEP of Diamond Light Source, UK [13]. Synchrotron X-ray imaging and tomography have been used extensively in recent years by many researchers to study the dynamics of solidification microstructures. Those particularly related to magnetic field solidification experiments can be found in [14], [15], [16], [17], [18].

The design of the magnetic coil system was based on the well-established series resonant circuit. It consists of a magnetic coil and a capacitor. A power amplifier (TOMCO Technologies BT00500-AlphaA-CW) receiving a low voltage, programmable wave signal from a function generator (Agilent 33220A) is used to drive the resonant circuit. Previous systems described in the literatures [8], [9], [10], [11], [12] used magnetic coils made from water-cooled copper tubing, with a low number of turns, similar to an induction heating coil. However, these systems are usually bulky, mainly due to the power supply unit and cooling system required, and therefore inconvenient to be moved around. In our system, the magnetic coil uses a high number of turns of fine copper wires instead. In addition, the system was developed according to the Helmholtz configuration, where a pair of magnetic coils were separated by a predefined distance. Such configuration provides a gap in between the magnetic coil pair, allowing X-rays to pass through, whilst the gap is small enough to maintain a uniform magnetic field within the central region.

In most industrial casting process, an alloy is melted and poured into a mould in the solidification processes. However, during a synchrotron X-ray experiment, this would not be technically feasible. Therefore, a furnace was designed and made to concentrically fit within the magnetic coils. A furnace system which incorporated a counter-wound design was built; the counter-wound design was utilised to reduce the effect of inductive coupling between the furnace heating coils and magnetic coils during operation. The design is discussed in more detail in Section 7.1. The furnace temperature was controlled by PID method.

The magnetic coil system is designed to operate at a frequency of 25 kHz. This particular frequency was selected according to the findings in [19], which demonstrated an optimal separation efficiency with the size of the sample being studied using this technique. However, the apparatus could be modified for other studies involving the uses of magnetic fields, for example:-

• Solidification processing of molten metal with DC or low frequency AC magnetic field.
• Magnetic particle imaging.

Hence the designed coil system could be exploited in other synchrotron X-ray experiments.

## 3 Design files

**Table:** 

Design folder name	File type	Open source license	Location of the file
Magnetic coil	CAD files	CC BY 4.0	http://dx.doi.org/10.17632/ymr2yhrvyn.2#folder-9e8a6810-5778-4fbc-a8ca-ae1ba549cb07
Cooling jacket	CAD files	CC BY 4.0	http://dx.doi.org/10.17632/ymr2yhrvyn.2#folder-9efac419-b723-4c5e-9066-b1075669abd8
Printed circuit board	Schematic	CC BY 4.0	http://dx.doi.org/10.17632/ymr2yhrvyn.2#folder-a5728ce5-f7d0-4e76-9c07-79bd0f075f30
Capacitor bank	CAD files	CC BY 4.0	http://dx.doi.org/10.17632/ymr2yhrvyn.2#folder-c27cf0d6-59e8-484a-abc6-124fc09b4919
Furnace	CAD files	CC BY 4.0	http://dx.doi.org/10.17632/ymr2yhrvyn.2#folder-dd8f8d50-e09a-45e2-9be2-d62252601673

The CAD files, with the exception of the magnetic coil, were produced using SolidWorks 2017 [20] and the schematic of the printed circuit board was produced using Cadence® Allegro® PCB design suite [21]. The Magnetic coil folder contains the CAD drawing of the magnetic coil designed by AGW Electronics Ltd. and permission had been granted by the manufacturer for the drawing to be published. The Cooling jacket folder contains the CAD drawings of the base plate and cylindrical cooling jacket half. The Printed circuit board folder contains the schematic layout drawing of the capacitor array. The Capacitor bank folder contains the CAD drawings of the enclosure and air vent. The Furnace folder contains the CAD drawings of the furnace, bracket, and lid.

## 4 Bill of materials

**Table:** 

Designator	Quantity	Unit cost (£ - GBP)	Total cost (£ - GBP)
Magnetic coil	1	1276.64	1276.64
Cooling jacket	1	68.98	68.98
Capacitor bank	1	1545.21	1545.21
Furnace	1	136.67	136.67

^1^ All prices exclude United Kingdom VAT of 20%.

^2^ Prices are approximate at time of purchase.

^3^ Any manufacturing done in-house was without the cost of labour.

The bill of materials listed above summarises the main components of the apparatus. For the full bill of materials, readers are referred to Appendix A. Supplementary data (File name: Bill of materials.xlsx). Note that other general laboratory equipment was not included in the bill of materials.

## 5 Design, fabrication, and assembly

### 5.1 Magnetic coil

In a conventional magnetic coil, a close-packed winding will unavoidably obstruct the X-ray path. Therefore, a pair of magnetic coils were designed, based on the Helmholtz-type configuration. This design allows the magnetic coils to produce a uniform magnetic field in the central region by separating the pair by a predefined distance to give an unobstructed viewing gap as discussed in more detail in Section 7.3.1. The manufacturing of the magnetic coils was made by AGW Electronics Ltd. to ensure the quality, reliability, and safety compliance of the magnetic coils. The specifications of the magnetic coil are listed in Table 1.

**Table 1:** Specifications of the magnetic coil.

Wire material	Enamelled copper
Insulation	Grade 2
Wire diameter (mm)	0.75
Sleeving material	Polytetrafluoroethylene (PTFE)
Winding inner diameter (mm)	60
Winding outer diameter (mm)	80
Winding height (mm)	27
Number of turns	300
Number of layers	10
Turns per layer	30
Bobbin material	Polyether ether ketone (PEEK)
Bobbin thickness (mm)	1.5

Owing to the high voltage requirements envisaged during the design stage, a 2 mm isolation margin tape (Type 44) between both sides of the bobbin was required. Between each successive layer of windings, a layer of polyester film electrical tape (Type 54) was used for additional insulation. After the completion of the winding process, the magnetic coils were vacuum impregnated with varnish to increase the durability of the coils.

### 5.2 Cooling jacket

**Fig. 2:**
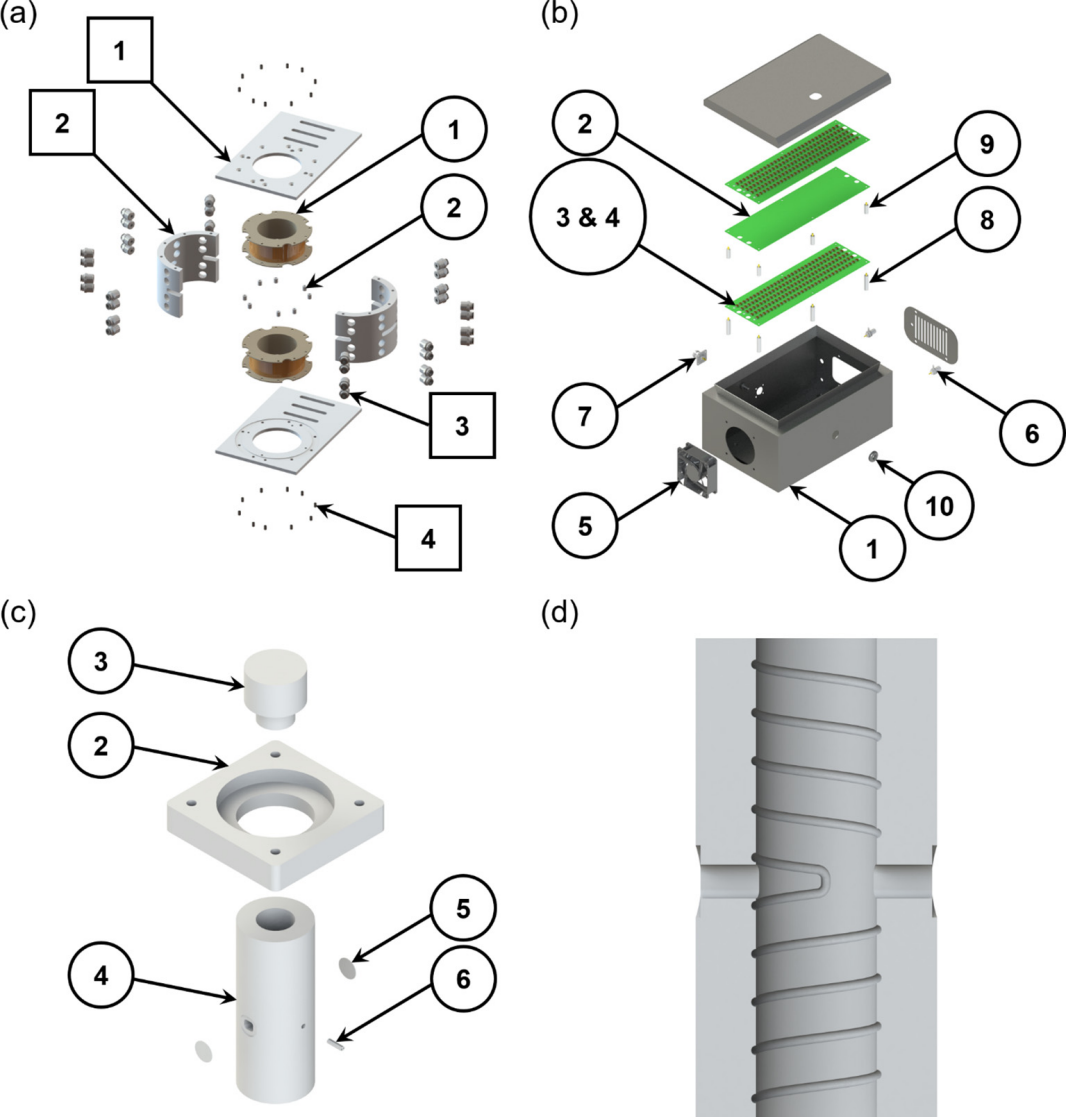
The exploded view CAD drawings of the (a) magnetic coils enclosed by the cooling jacket (Circular and square balloons represent the numberings for the magnetic coil and cooling jacket, respectively), (b) capacitor bank, and (c) completed furnace (Electrical resistance wire and thermocouple are not shown in the drawing). (d) Cross-sectional view of the furnace, illustrating the profile of the counter-wound windings. [Note: Balloons represent the component numbering corresponding to the full bill of materials]

The cooling jacket is comprised of four separate parts (Fig. 2a); two base plates and two halves of the cylindrical cooling jacket. The two base plates were cut to the desired size (200 × 130 × 5 mm), followed by the drilling of holes and alignment slots through the base plates. A 1 mm deep recess (120 mm OD and 100 mm ID) was bored into the surface, where the cooling jacket will be securely located for concentricity.

The cooling jacket was machined from a nylon rod (sectioned and turned down to the desired dimensions of Ø120 × 77 mm). A Ø100 mm hole was then bored through the centre of the work piece to create the inner chamber. Four series of eight through holes, evenly distributed radially, were drilled and tapped, each series spaced 15 mm apart axially, except in between the inner two series where there was a spacing of 27 mm, allowing for a viewing slot. The tapped and drilled holes were for the attachment of pneumatic adapter fittings. The viewing slots, of which there were four, were 7 mm in height and were along the mid-plane of the work piece, at a right angle to one another. The slots served two purposes; one was to allow for video imaging capability (e.g., visible light, laser, X-ray beam, or neutron beam), and the other was to allow the leads from the magnetic coils to come out from the enclosed unit. Additional tapped holes were drilled at the top and bottom of the cooling jacket for threaded inserts to be screwed in. In order to split the cooling jacket in half, two narrow blind depth slots were cut axially along the surface at a 180° from one another. A craft knife was then used to cut through the remaining material to form the halves.

### 5.3 Printed circuit board (PCB)

The resonant capacitors were made from an array of commercial surface mount capacitors, which were connected electrically in parallel and series (Fig. 2b). Variations in capacitances within each series chain may cause voltage imbalances along each chain, and then overstress the individual capacitors beyond their absolute maximum ratings. Simulations were carried out to determine the electrical stress by assuming the worst case scenarios. The results showed that even in the worst case, the voltage across any individual capacitor would not be exceeded.

Given the capacitors were surface mounted, a regular printed circuit board (PCB) was considered as the optimum choice for creating the circuits. The stages for PCB production were:

1. Generating the electrical schematic for one PCB.
2. Generate a netlist of components connectivity.
3. Layout the PCB using the netlist connectivity.
4. Output the PCB manufacturing data.
5. Order the PCB from a commercial PCB fabrication service.
6. Order the PCB component assembly service.

Steps 1–4 were carried out using the Cadence® Allegro® PCB design suite [21], which is a complete software package for generating electronic schematics through to PCB manufacturing data.

Steps 5–6 were chosen over an in-house build to ensure that the products were of sufficient quality and consistency to endure the electrical and thermal stresses reliably.

### 5.4 Capacitor bank

The physical dimensions (90 × 292 × 3.95 mm) of the PCBs (including the capacitors) heavily influenced the selection of an enclosure for the capacitor bank. The PCB physical dimensions were driven by the requirement to have sufficient creepage and clearances for the high voltages that would be present across each PCB. Three PCB assemblies were connected electrically in parallel and mechanically positioned above each other (Fig. 2b). Sufficient clearance between the boards allowed cooling air to pass over the assemblies. This also necessitated a cooling fan and exhaust to be fitted to the enclosure. Therefore, an electrical steel wall box (150 × 300 × 200 mm) was chosen for the application. Holes were drilled in the enclosure to accommodate the required electrical and mechanical fittings. An array of slots was also drilled on the cable gland plate to provide an outlet for the forced air cooling from the DC fan.

### 5.5 Furnace

The furnace was specially designed to fit in between the sample and the magnetic coils (Fig. 1). This particular system was intended to be used for the melting of aluminium alloys with melting temperatures below 850°C, hence electrical resistance heating wires, capable of withstanding temperatures up to 1150°C was chosen for the application. The heating element was made of nichrome (Ni-Cr) wire which is currently the most typical heating element used in electrical heating devices.

According to the formula for electrical resistivity, the resistance of a wire is directly proportional to the specific electrical resistance of the material and its length, and inversely proportional to its cross-sectional area (*R* = *ρl/A*). Thus, the heating element is typically constructed in a helix to achieve a large length-to-area ratio for it to be operated efficiently. Ten metres of Ø0.25 mm Ni-Cr wires was wound in order to achieve the desired helix heating element (3.25 mm OD) with a length of straight wire, ~10 mm, at the midpoint of the work piece, along the axis to minimise any potential inductive coupling effects. This design is discussed in more detail in Section 7.1. Additional straight wire sections at the ends were left intact for the purpose of being wire leads for electrical connections.

**Fig. 3:**
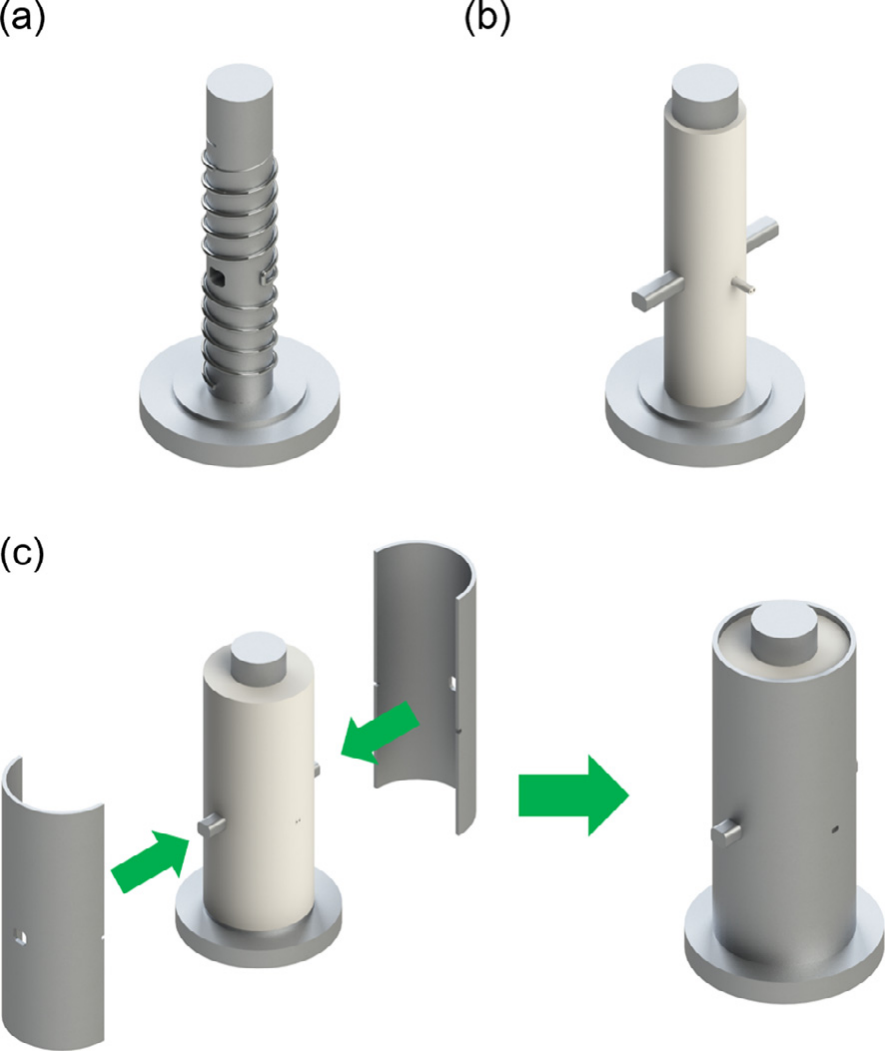
CAD drawings, illustrating the steps to manufacture the furnace. (a) The heating coil was wound around the mandrel following the machined groove profile. (b) Layers of ~2–3 mm thick mouldable ceramic insulation being applied in succession after air-dried in an oven. (c) The two die halves used to compress the insulation material at later stages of the moulding process.

A custom designed mould (Fig. 3) was machined to accommodate the profile of the furnace windings, an aperture, and also a ceramic thermocouple sheath. Two sets of winding profile were machined onto the Ø25 mm mandrel of the mould, at opposite directions, clockwise/counterclockwise, with a straight section at the midpoint to connect the two profiles. Such design allowed the heating coil windings to achieve a counter-wound configuration, which is discussed in more detail in Section 7.1. An oblong hole with the dimensions of 8 × 7 mm (horizontal × vertical), for the aperture, was machined in between the two sets of helix profiles and on the two cylindrical die halves. A small slot was also made in the die halves, perpendicular to the aperture, to accommodate a twin-bore ceramic thermocouple sheath. A key was also machined, to tightly fit into the oblong hole, to prevent any blockage to the aperture during the moulding operation.

The steps to manufacture the furnace is illustrated in Fig. 3. The heating coil was wound around the mandrel following the machined groove profile, with the straight wire section of the heating element aligning with the straight section of the machined profile (Fig. 3a). Mouldable ceramic insulation (Fiberfrax® Mouldable 120) was then applied in layers, with forced air-drying in an oven, at a temperature of ~150°C, between application of successive layers until a wall thickness of ~12.5 mm was achieved. Each layer of insulation material was ~2–3 mm thick (Fig. 3b). At later stages, the two die halves were used to compress the insulation material, as illustrated in Fig. 3c, due to the tight clearance between the bobbin of the magnetic coils and the outer wall of the furnace. Once the moulding process was completed, a final drying process to remove any excess moisture was carried out, where the furnace was removed from the mould and was heated up to ~750°C for a prolonged period (>1 h). The final drying step also helps in hardening the heating wire whilst securing the shape into the insulation. Furthermore, a bracket for the furnace was also machined from a Duractec® 750 machinable ceramic sheet. This allowed for holes to be machined onto the ceramic, for the furnace to be mounted securely onto additional mounting brackets. A lid to prevent the heat loss from the top of the furnace was also machined from a Ø40 mm Duratec rod. A 0.5 mm thick quartz disc was also mounted on both sides of the aperture to prevent excessive convection heat loss via the aperture. Fig. 2c shows the exploded view CAD drawing of the completed furnace and Fig. 2d is a cross-section of the furnace, illustrating the profile of the counter-wound windings.

**Fig. 4:**
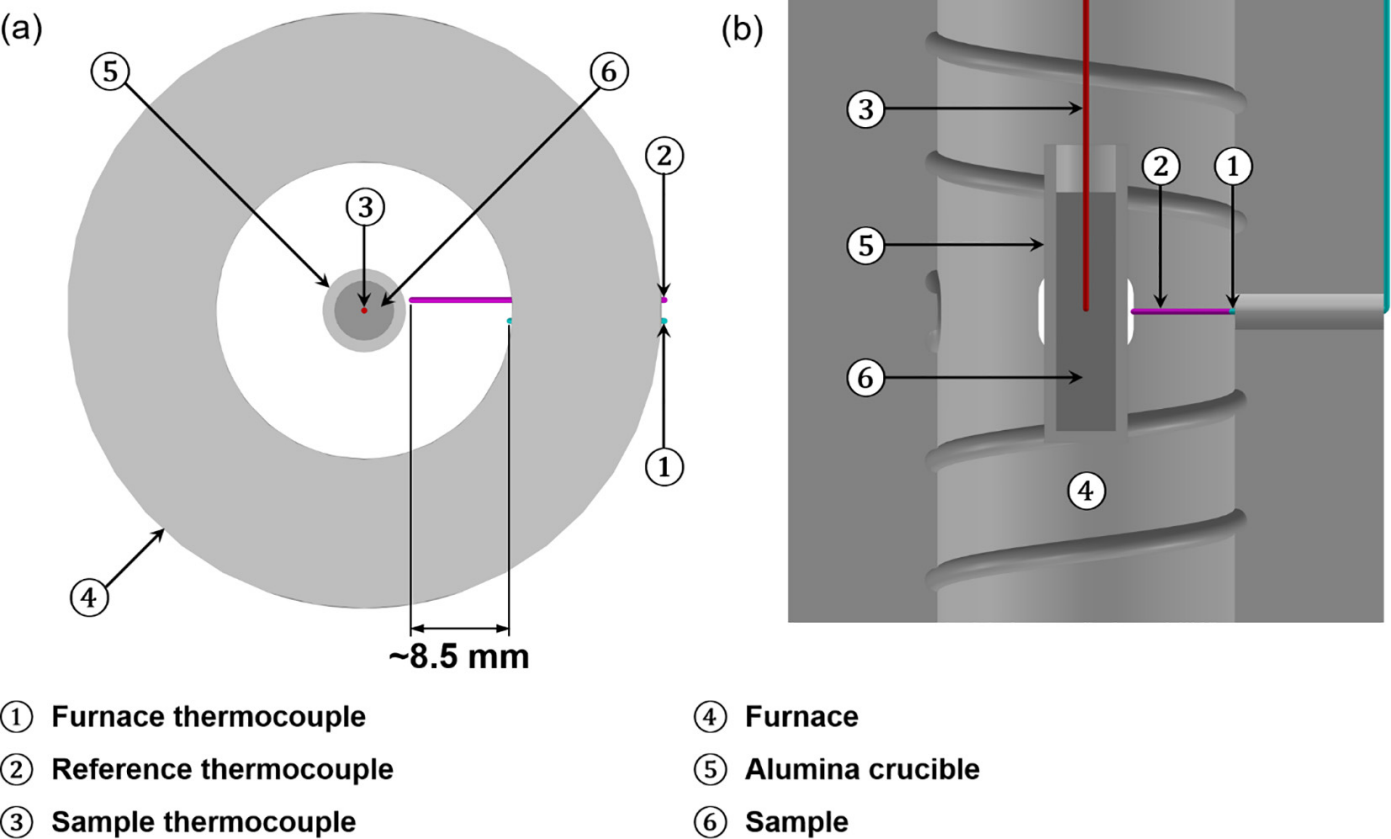
The (a) top and (b) cross-sectional view of the furnace, illustrating the position of the thermocouples and the sample inside the furnace (The extension piece, which holds the crucible, is not shown in the drawing).

Two Ø0.5 mm K-type thermocouples were inserted into the twin-bore ceramic thermocouple sheath for temperature monitoring, controlling, and recording (Fig. 4); one was positioned close to the inner wall of the furnace (No. 1 thermocouple in Fig. 4), while the other was extended further out by ~8.5 mm (No. 2 thermocouple in Fig. 4), to be in a location as close as possible to the alumina crucible (the metal alloy sample holder). No. 1 thermocouple was used to monitor and control the temperature of the heating coil, while No. 2 thermocouple was used to monitor and record the crucible (metal alloy) temperature. No. 2 thermocouple was not directly placed into the metal alloy to avoid any possible entanglement of the thermocouple when the sample is rotated during tomography acquisitions. The sample thermocouple (No. 3 thermocouple in Fig. 4) is only used for *ex-situ* experiments and temperature calibration runs, where the sample is not rotated.

During the experiments, the sample (Ø5 × 20 mm) is centrally positioned within the field of view of the furnace aperture and is contained within an alumina crucible (5 mm ID × 25 mm with a wall thickness of 1 mm), as shown in Fig. 4. The extension piece, which holds the crucible, is also made from the same alumina material to withstand the high temperatures within the furnace chamber. A stainless steel adapter was also machined for the extension piece to sit securely within, whilst also allowing the adapter to be mounted onto additional fixtures. The sample holder assembly is then mounted onto linear translation and rotation stages. The sample is then raised into the furnace chamber from the bottom opening and aligned to the aperture using the linear translation stages.

### 5.6 Magnetic coil system

The magnetic coil system consists of four parts: (1) magnetic coils, (2) capacitor bank, (3) RF power amplifier (TOMCO Technologies BT00500-AlphaA-CW), and (4) function generator (Agilent 33220A). A parallel configuration was employed for the magnetic coil pair and is discussed in more detail in Section 7.2.1. The capacitor bank, which acts as an impedance cancellation device, is then connected to the magnetic coil pair to create a series resonant circuit. Readers are referred to Section 7.2.2 for further details. The ideal separation distance between the magnetic coil pair, to achieve a uniform magnetic field, was determined through simulation and verified through experiments, and is discussed in more detail in Section 7.3.1. As a result, the magnetic coils were separated by spacers of 7 mm in height, by taking into account the thickness of the magnetic coil bobbins and insulating material. The magnetic coil pair were then contained within the cooling jacket. The capacitor bank is then connected to the amplifier via an RF coaxial cable. A function generator, which provides the driving signal, is connected to the amplifier via a BNC cable.

**Fig. 5:**
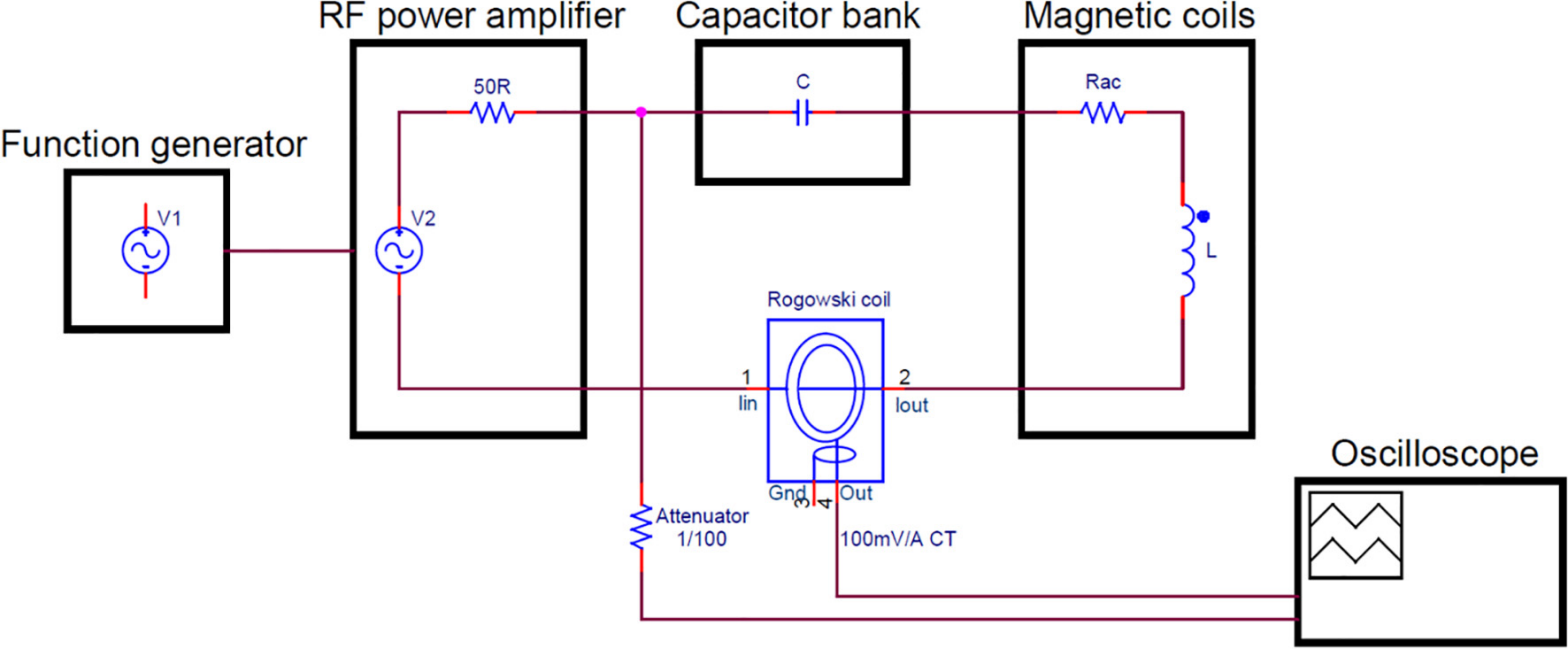
A simplified schematic diagram of the magnetic coil system.

A current probe (GMC I-PROSyS ACP 2005/2), based on the Rogowski coil principle, was utilised to monitor the output current via an oscilloscope (Tektronix TDS3012C). The output cable of the current probe was extended to 15 m in length for being used in a synchrotron experimental hutch. The modified current probe was tested and verified that there were no negative effects on the output measurements. In addition, the output voltage from the amplifier was also recorded via BNC through a 20 dB attenuator and linked to the oscilloscope, which was securely mounted onto the enclosure of the capacitor bank. A simplified schematic diagram of the magnetic coil system is represented in Fig. 5.

### 5.7 Furnace system

The furnace system consists of three parts: (1) furnace, (2) temperature control unit, and (3) PC. The furnace was connected directly to the temperature control unit, which is linked to a PC. The in-house built PID temperature control unit is used to control the temperature of the heating wire using No. 1 thermocouple in Fig. 4. It provided the feedback signal to the PID controller, which automatically regulates the heating power to compensate the difference between the desired and the measured temperature. The reference temperature, from No. 2 thermocouple in Fig. 4, is either connected to a temperature data logger (Pico Technology TC-08) for *ex-situ* laboratory-based experiments, or directly into Beamline I12′s internal data logging system.

### 5.8 Safety

#### 5.8.1 Exposure to electromagnetic radiation

The apparatus was designed for being used on Beamline I12 - JEEP of Diamond Light Source, UK. The high operating X-ray energy range (53–150 keV) of Beamline I12 was an important consideration for designing any equipment to be used in such harsh radiative environment, particularly the material selection. The bobbin of the magnetic coils is made out of PEEK. PEEK was chosen over other conventional polymers, due to its inherent resistant to ionising radiation. Apart from that, the majority of the cooling jacket is made from nylon, which possesses acceptable resistance to X-ray damage. Electronic components are also kept to a minimum within the circuitry to avoid potential radiation damage.

The presence of an alternating magnetic field also contributed to the material selection process. Polymeric materials, i.e., PEEK and nylon, were used to avoid any potential influence on the magnetic field. With the furnace being in direct exposure to the alternating magnetic field, it was crucial that the furnace was not constructed with any conducting materials, with the exception of the electrical resistance wire. Therefore, mouldable and machinable ceramic were the preferred materials for the construction of the furnace.

The maximum magnetic flux density achieved within the central region of the magnetic coils with the current setup was ~10 mT. The intensity of the magnetic flux density rapidly depreciates as the distance from the centre of the magnetic coils increases. The simulated intensity of the magnetic flux density at 100 mm from the centre of the magnetic coils was shown to be ~0.46 mT. The stray magnetic field at such a distance was considered negligible.

#### 5.8.2 High voltage

The series resonant circuit is a very useful technique to achieve minimum circuit impedance. In a perfectly resonating circuit, the reactive components can be depicted as being a short circuit (*X_L_* = *X_C_*). With only the real component (*R_AC_* = *Z*) remaining, according to Ohm’s Law (*I* = *V/Z*), this allowed a higher level of current to pass through the magnetic coils. However, the magnitude of the reactive voltages across both the inductor and capacitor was amplified to many times larger, in the kilovolt range, than the supplied voltage delivered from the amplifier. The high-risk portion of the circuit established is between the capacitor bank and the magnetic coils (Fig. 5), where the voltage amplification occurred. Therefore, these two components are kept at a relatively short distance to each other, with the leads from the magnetic coils connected directly to the capacitor bank. The highest potential difference exists between the leads of the magnetic coil, hence were insulated with a PTFE sleeving of 0.3 mm wall thickness. The next biggest potential difference exists in between the winding layers, where the polyester film electrical tape was added for additional insulation. Both the capacitor bank and magnetic coils are also contained within an enclosed unit, i.e., PCBs in the electrical steel wall box, and magnetic coils in the cooling jacket. As a result, any potential physical contact with the high voltage components is prevented.

#### 5.8.3 Temperature

With the furnace being placed within the magnetic coils (Fig. 1), the surface of the magnetic coil bobbin is highly prone to damage by the radiative heat emitting from the furnace outer walls. Therefore, another reason for choosing PEEK as the material for the magnetic coil bobbin is due to its excellent temperature properties, as compared to other polymeric materials. PEEK has a continuous service temperature of up to ~250°C with a melting temperature of ~343°C [22]. The cooling jacket was less susceptible to thermal failure, hence nylon, which is readily available and a cheaper alternative, was used.

## 6 Operation instructions

### 6.1 Magnetic coil system

The operation of the magnetic coil system is relatively straightforward. First, the desired amplitude of the input voltage and operating frequency is set on the function generator. When both the function generator and amplifier are enabled, a low-level sinusoidal signal from the function generator is transmitted into the amplifier, which amplifies the signal by a fixed gain. The amplified signal is then output from the amplifier and into the magnetic coil system.

### 6.2 Furnace system

The temperature of the furnace is monitored and controlled through the software, Platinum Configurator [23], developed specifically for the PID controller used in the temperature control unit. The Platinum controller offers a convenient Ramp and Soak control, which provides users with the ease of controlling the heating/cooling temperature profiles. The desired temperatures, ramping and soaking time are set respectively before operating the furnace. The temperature data are continuously logged, in the background, during the operation of the furnace system.

### 6.3 Safety

#### 6.3.1 Temperature

The magnetic coils will heat up rapidly during operation due to the exponential relationship with the current passing through the system. Readers are referred to Section 7.3.2 for further details. Compressed air is continuously fed through the pneumatic adapter fittings to assist in the dissipation of heat from the magnetic coils during the operation to prevent the magnetic coils from overheating.

The temperature (>100°C) at the outer walls of the furnace was also identified as a hazard. However, the majority of the furnace is always surrounded by the magnetic coils during operation, which acts as a barrier for any possible physical contact with the furnace. Therefore, the risk of having physical contact with the furnace is minimal.

#### 6.3.2 General

Both the function generator and amplifier are disabled when not in use. As another precaution, the PID controller is left in a “standby” state and furnace is left to cool to ambient temperature when not in operation.

## 7 Validation and characterisation

### 7.1 Furnace-magnetic coils coupling

The inductive coupling between the furnace heating coils and the magnetic coils while in operation posed a potential problem for the configuration of the apparatus. According to Faraday’s law of induction [24], the primary winding which receives an alternating current will generate an alternating magnetic field. As a result, an alternating electromotive force (EMF) will be induced across the secondary winding that was wound around the same core. In this application however, the furnace (secondary winding) was enclosed within the magnetic coils (primary winding) and was air-cored. As the magnetic coils had a much greater number of windings as compared to the furnace (*N*_*P*_ > *N*_*S*_), the apparatus would innately behave as a step-down transformer.

It was hypothesised that a furnace which has two sets of winding in opposite directions, connected in series, would alleviate this inductive coupling effect. In theory, the induced EMF picked up by the clockwise windings would be opposed by that of the counterclockwise windings, or vice versa, hence resulting in a net EMF of 0 V. It was essential that the windings were connected in series, otherwise the two individual furnace windings wound in different directions will independently be affected by the alternating magnetic field, similar to the coupling effect of a conventional-wound furnace, which was undesirable. Besides that, the series connection between the two sets of windings had to be made out of a straight wire and not coiled, otherwise the short coiled section would also couple with the alternating magnetic field as it would be aligned with the direction of the magnetic field.

An experiment was conducted to confirm this hypothesis by having the two separate furnaces (conventional- and counter-wound furnace) positioned within the magnetic coils, similar to the experimental setup. An inductance analyser (Wayne Kerr 3225B) was used to drive an excitation voltage of 10 V_AC_ at a frequency of 30 kHz across the magnetic coils. The waveforms for the input voltage into the magnetic coils and the induced EMF from the furnace were recorded by using an oscilloscope (LeCroy Waverunner LT354). The results provided a clear difference between the two furnaces, whereby the conventional-wound furnace was picking up an induced EMF of 60.06 mV_RMS_, while the counter-wound furnace was only picking up 5.65 mV_RMS_. From comparison, a reduction by an order of magnitude was evident from the results, however as mentioned previously, the net EMF should theoretically be 0 V. As the furnace was made in-house, susceptibility to variations in the windings were unavoidable. Besides that, additional partial turns in the windings or the imperfect alignment between the alternating magnetic field and the windings may also result in the picking up of residual voltage.

### 7.2 Electrical characterisation

#### 7.2.1 Series vs parallel magnetic coils configuration

The magnetic coil pair was tested in the two simplest configurations, i.e., series and parallel. A frequency sweep (20 Hz–500 kHz) was performed with the inductance analyser (Wayne Kerr 3225B) driving an excitation voltage of 1 V_AC_ into the magnetic coil pair. Table 2 shows the values acquired at the desired operating frequency of 25 kHz for both series and parallel magnetic coils configuration. For the full characterisation of the frequency sweep, readers are referred to Appendix A. Supplementary data (File name: Series-Parallel configuration.xlsx). Note that the tests were carried out with only the magnetic coils.

**Table 2:** Measured electrical characteristics of the magnetic coils connected in series and parallel.

Electrical Properties	Series	Parallel
*R_DC_* (Ω)	5.163	1.29
*R_AC_* (Ω)	76.7	19.2
*L* (H)	15.47 × 10^−3^	3.867 × 10^−3^
*Z* (Ω)	2431	607.8

^1^Values for *R_AC_*, *L*, and *Z* shown were measured at the desired operating frequency of 25 kHz.

According to Table 2, the AC resistance (*R_AC_*) of the magnetic coils for both configurations are ~15× higher as compared to its DC counterpart. Numerous factors may have contributed to the significant increase in the *R_AC_*, e.g., skin effect and proximity effect, and were taken into consideration during the design stage. Therefore, *R_AC_* here is the summation of *R_DC_* (DC resistance), *R_SKIN_* (Skin effect), and *R_PROX_* (Proximity effect), which contributed to the total power dissipation of the magnetic coils. These effects will be discussed in more detail below.

As the frequency of an alternating current (AC) increases, the current density tends to increase at the surface of the conductor, and decreases exponentially towards the centre of the conductor. This phenomenon is known as the skin effect, and the depth from the surface to which the current density falls to about 37% (*1/*exp) of the surface current density is known as the skin depth layer [25]. This layer is highly attributed to the frequency of the system; the higher the frequency, the narrower the skin depth layer. The skin depth for copper conductors at the frequency of 25 kHz is ~0.4126 mm. Therefore, in order to minimise the effect of skin depth to allow the entire cross-sectional area of the conductor to be used, the radius of the copper wire chosen for the magnetic coil windings was ~0.375 mm. In effect, the skin depth is now ~11% larger than the effective cross-sectional area of the copper wire used [26].

The proximity effect occurs due to the closely wound windings in the magnetic coil. When an AC flows through the wires, an alternating magnetic field will be generated which induces eddy currents in the adjacent wires. As a result, the overall current distribution within the windings will be altered. The current distribution will tend to concentrate on the surface of the wire where the magnetic field intensity is most intense, with little or no distribution on the outside surfaces where there is a weaker field [25]. As the name implies, the distance between the wires could be spaced further apart in order to reduce this effect. A thicker insulation layer over the copper wire would aid in reducing the effect, however this also further reduces the achievable magnetic field intensity.

The total number of winding layers could potentially be the major contributor to the increase in *R_AC_* in both configurations. The correlation between the ratio of AC and DC resistance (*R_AC_*:*R_DC_*,), and number of winding layers had been well established in Dowell’s work on eddy current losses in windings using sinusoidal waveforms [27]. According to Dowell [27], this ratio increases with increasing number of winding layers. Although Dowell’s approximation might not be suitable for the field geometry in this particular application, it provided a good indication on the eddy current losses of multi-layer windings, and how it contributed to the AC resistance.

Although the *R_AC_* was increased by a substantial amount due to the aforementioned effects, the total impedance (*Z*) of the circuit was mainly contributed from the inductive reactance (*X_L_* = *2πfL*), evident from Table 2. The inductance for both configurations was almost constant, hence its inductive reactance increased linearly with frequency. Owing to the high impedance of the serially connected magnetic coils configuration, the parallel configuration was considered as the better option.

#### 7.2.2 Series resonant configuration

The same frequency sweep was also performed for this configuration. Table 3 shows the values acquired at the desired operating frequency of 25 kHz for the series resonant configuration. For the full characterisation of the frequency sweep, readers are referred to Appendix A. Supplementary data (File name: Series resonant configuration.xlsx). Note that the measured inductance had a negative value. The inductance analyser works by measuring the voltage across and current passing through the circuit, and then calculating the impedance (*Z*) and phase angle (*θ*). All the other AC measurement parameters (e.g., *L*, *C*) are then calculated from *Z* and *θ* using the standard equation. With the inductance being a negative value, this meant that the series resonant circuit was leaning towards being capacitive at a frequency of 25 kHz.

**Table 3:** Measured electrical characteristics of the series resonant circuit.

Electrical Properties	Series Resonant
*R_AC_* (Ω)	19.795
*L* (H)	−57.36 × 10^-6^
*C* (F)	706.5 × 10^-9^
*Z* (Ω)	21.75

^1^Values shown were measured at the desired operating frequency of 25 kHz.

In the previous sub-section, the total impedance for a parallel configuration (Table 2) was 607.8 Ω. This would imply that a power supply capable of generating ~608 V_RMS_ would be required in order to generate a current of 1 A_RMS_ passing through the circuit. In effect, only 0.5 A_RMS_ will pass through each magnetic coil due to the parallel configuration. Thus in order to supply a much higher current to generate a more intense magnetic field, a larger power supply unit, many kilowatts (kW), would be required. This would be impractical as the system was designed for its portability, e.g., to be used in a laboratory-based environment or in synchrotron facilities around the world.

As a result, a series resonant configuration was chosen and built to eliminate the reactive component of the system. Ideally, the capacitive reactance (*X_C_*) cancels out the inductive reactance (*X_L_*) for a perfectly resonating system, leaving behind only the real component (*R_AC_*). The resonating capacitors contributed an infinitesimal *R_AC_* due to the equivalent series resistance (ESR), evident from the marginal increase in *R_AC_* in the series resonant configuration (Table 3) as compared to the parallel configuration (Table 2). Due to the very narrow bandwidth and high quality factor (Q factor) of a resonant circuit, even slight changes in the inductance or capacitance would throw the system off resonance. The resonating capacitors, like the magnetic coils, will tend to heat up during the operating duration, and this may cause a drift from the nominal capacitance due to temperature and voltage stresses incurred. However, the resonating capacitors chosen for the application are highly resistant to such capacitance fluctuations and were mainly attributed to the excellent dielectric (C0G) within the capacitors which has a temperature coefficient of 0 ppm/°C and a tolerance of ±30 ppm/°C [28]. This excellent temperature coefficient resulted in the capacitance fluctuations being almost negligible.

### 7.3 Magnetic field characterisation

#### 7.3.1 Relationship between the separation distance and uniformity of magnetic field

The uniformity of the magnetic field along the symmetrical axis of the magnetic coils is important to this particular application, and was attributed to the separation distance between the magnetic coil pair. The optimal separation distance was determined through simulation, and verified through experiments and theoretical calculations.

**Fig. 6:**
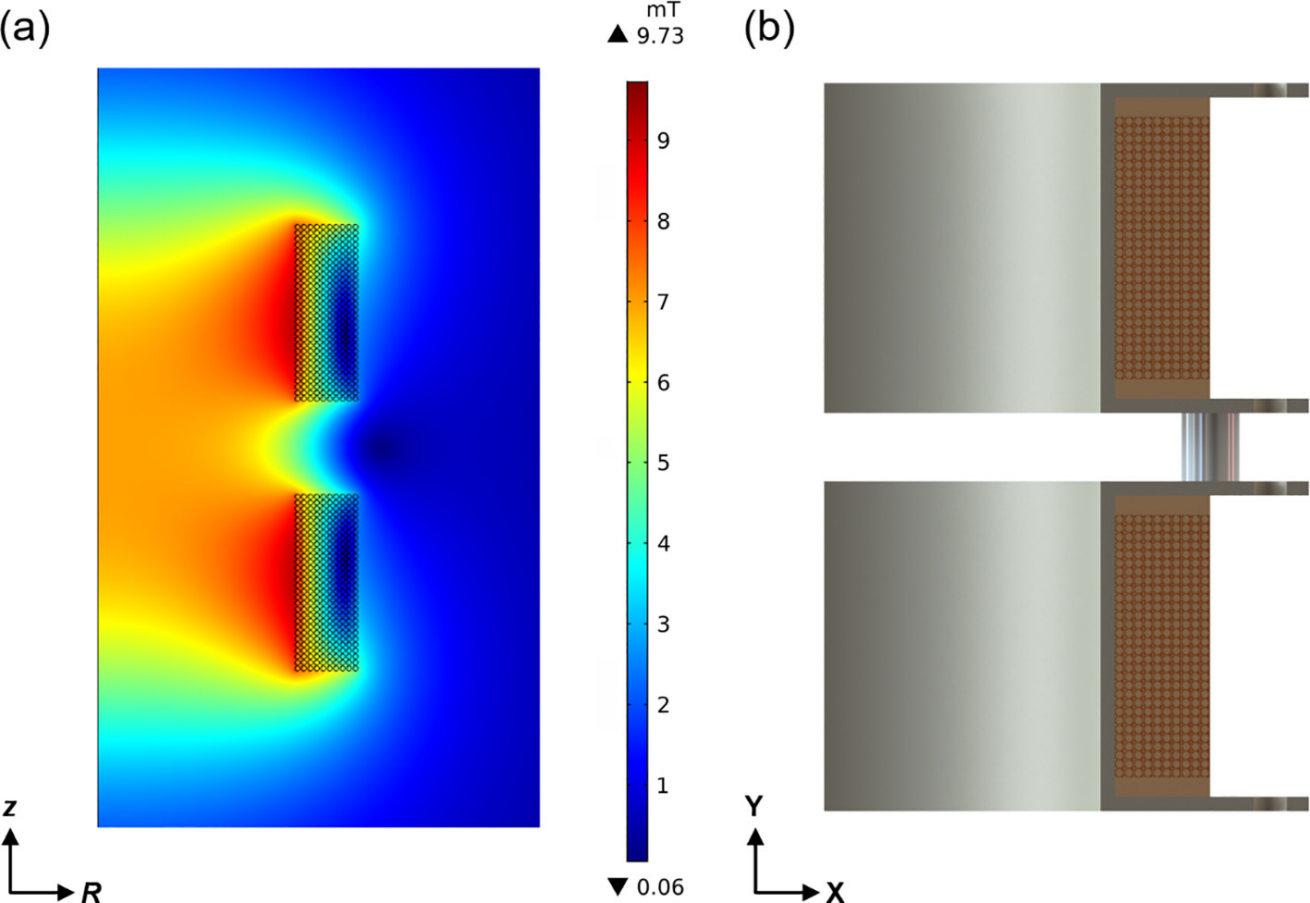
The (a) simulated 2D axisymmetric model and (b) CAD drawing of a quadrant section of the magnetic coils.

Comsol Multiphysics® v5.4 software [29] was used to perform the modelling and simulation of the magnetic coil pair with the AC/DC module [30]. Since the geometry of the magnetic coils is symmetrical along the common axis, a 2D axisymmetric model was used to reduce the size of the computational domain. The bobbin of the magnetic coils, spacers, and mechanical fasteners were made from non-magnetic material, hence were not included in the simulation model. The simulated 2D axisymmetric model and CAD drawing of a quadrant section of the magnetic coils are shown in Fig. 6. The dimensions of each winding were accurately modelled according to the CAD drawing provided by the manufacturer (AGW Electronics Ltd.). All of the simulations were performed with a current of 1 A_RMS_ passing through each coil.

**Fig. 7:**
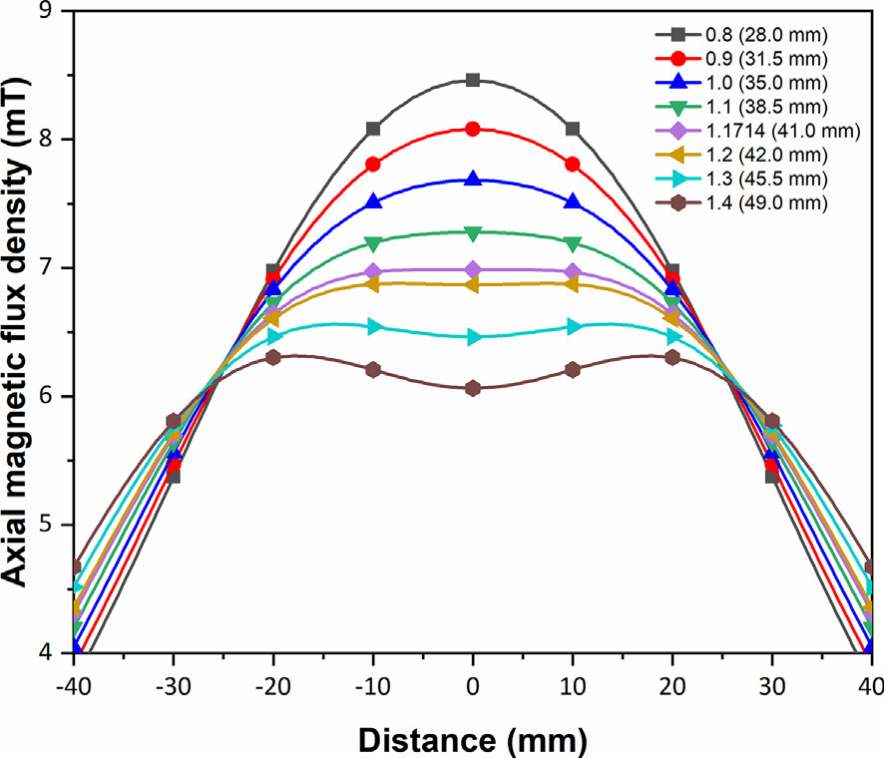
Simulated magnetic field uniformity at varying magnetic coil mid-plane separation distances.

Fig. 7 represents the correlation between the separation distance and the mean radius of the magnetic coils (*R_MEAN_*). According to the Biot-Savart Law [31], the optimal mid-plane separation distance between the magnetic coils is equal to its mean radius (*x:R_MEAN_* = 1:1), similar to a Helmholtz-type configuration. However, the simulated results did not agree completely with the Biot-Savart Law as it was clearly demonstrated in Fig. 7. To demonstrate a uniform magnetic field, the simulated curve has to reach a plateau and remain flat at the central region, but a discernible crest formed when the *x/R_MEAN_* = 1.0, which indicated that the magnetic coils were spaced too close together. Conversely, a trough began to form when the magnetic coils were spaced too far apart from each other. For this particular magnetic coil configuration, it was determined that the optimal mid-plane separation distance between the magnetic coils and its mean radius was 1.1714 (*x/R_MEAN_* = 1.1714). Geometrical aspects of the magnetic coil windings (Fig. 6) were the most likely cause of such discrepancies.

**Fig. 8:**
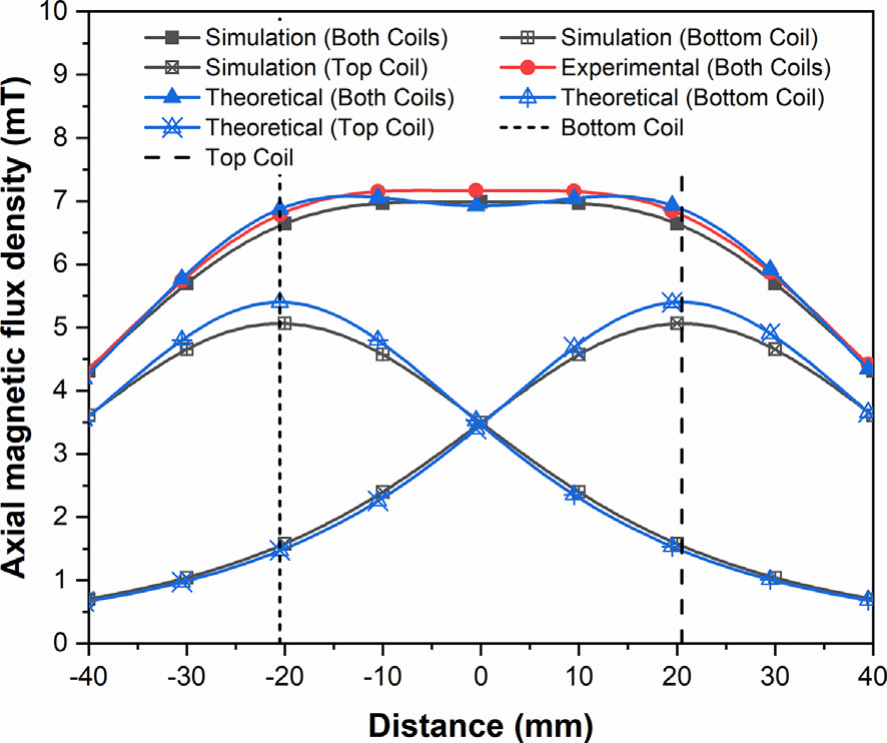
Comparison between simulated, experimental, and theoretical axial magnetic flux density.

With the separation distance defined, an experiment to measure the axial magnetic flux density was carried out. A direct current (DC) power supply (TTi EL302D) supplied a constant 2 A current into the magnetic coils and the axial magnetic flux density measurements were recorded by using a gaussmeter (Hirst Magnetics GM08) with an axial probe (Hirst Magnetics AP002) along the symmetrical axis of the magnetic coils. The experimental measurements were repeated three times and the averaged results from those three measurements are represented in Fig. 8. DC was used in this experiment rather than its AC counterpart as DC provided a more stable supply of current and minimised potential Joule heating, resulting in DC magnetic flux density measurements being acquired. The different current type used here does not impair the credibility of the results as the instantaneous current for both DC and AC, e.g., 1 A, will produce the same magnetic flux density.

The theoretical calculations were numerically solved by using the Biot-Savart Law [31]. For a pair of identical coils separated by an arbitrary distance, the total magnetic flux density can be defined as the superposition of the magnetic flux density contributions from each coil at each discrete position along the symmetrical axis of the magnetic coils, respectively, and can be expressed as:

(1) $$B\left(z\right)=\frac{\mu_{0}NI{R_{\mathrm{MEAN}}}^{2}}{2}\left\{\frac{1}{{\left[{R_{\mathrm{MEAN}}}^{2}+{\left(z+\frac{x}{2}\right)}^{2}\right]}^{\frac{3}{2}}}+\frac{1}{{\left[{R_{\mathrm{MEAN}}}^{2}+{\left(z-\frac{x}{2}\right)}^{2}\right]}^{\frac{3}{2}}}\right\}$$

where *µ_0_* is the permeability of free space (H/m), *N* is the number of turns in each magnetic coil, *I* is the current passing through the magnetic coils (A), *R_MEAN_* is the mean radius of the magnetic coil windings (m), *z* is the position along the symmetrical axis of the magnetic coils (m), and *x* is the mid-plane separation distance between the magnetic coil pair (m).

The comparison between the simulated, experimental, and theoretical axial magnetic flux density profiles are plotted in Fig. 8. The two vertical dashed lines represent the position of the top and bottom coils (*z* = ±20.5 mm). According to Fig. 8, both the simulated and experimental profiles showed a similar trend and agreed well. However, the theoretical profile demonstrated a trough, which implied that the magnetic coil pair were spaced too far apart from each other. As previously mentioned, the physical geometry of the coil windings, which in this case was far from ideal, would produce minor discrepancies. Numerous factors, e.g., cross-sectional area and arrangement of the windings, were not considered in Eq. (1). Although the mean radius (*R_MEAN_*) of the magnetic coil windings were used, some uncertainties within the theoretical calculations would still exist [32]. Nonetheless, the theoretical calculations were able to provide a decent approximation in terms of the separation distance.

The measure of normalised uniformity of the magnetic flux density can be defined as:

(2) $$\varepsilon =1+\frac{B\left(z\right)-B\left(0\right)}{B\left(0\right)}$$

where *B*(*z*) is the magnetic flux density at any position along the symmetrical axis of the magnetic coils and *B*(0) is the magnetic flux density at the centre of the magnetic coils (*z* = 0).

**Fig. 9:**
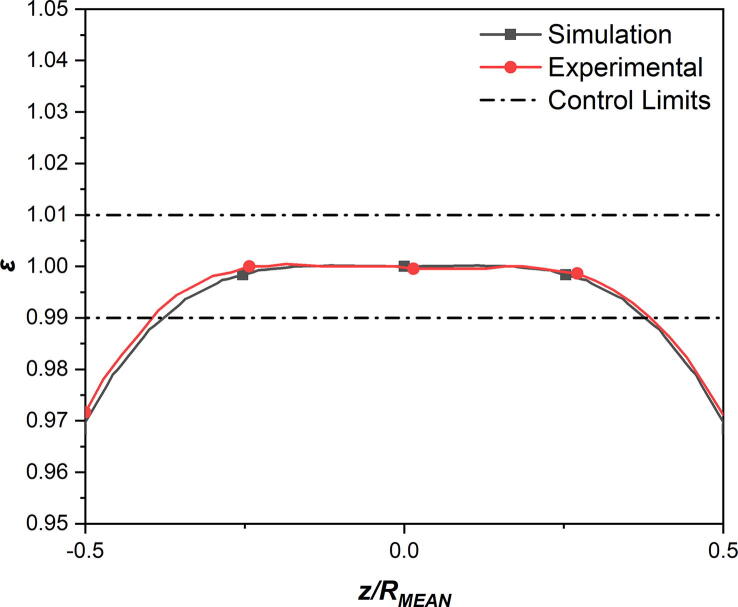
Normalised axial magnetic flux density for the simulated and experimental results displaying excellent uniformity.

Fig. 9 shows the simulated and experimental profiles of the normalised axial magnetic flux density, which displayed an excellent agreement. The theoretical profile had been omitted for clarity, due to discrepancies as mentioned previously. The measure of uniformity in a magnetic field depends mainly on the requirements of the application. For this particular application, a variance in the magnetic flux density of ±1% (Fig. 9) was considered to be acceptable. According to Fig. 9, the central region which displayed the uniformity of magnetic field within the control limits corresponded to ~26 mm in length in the real space.

**Fig. 10:**
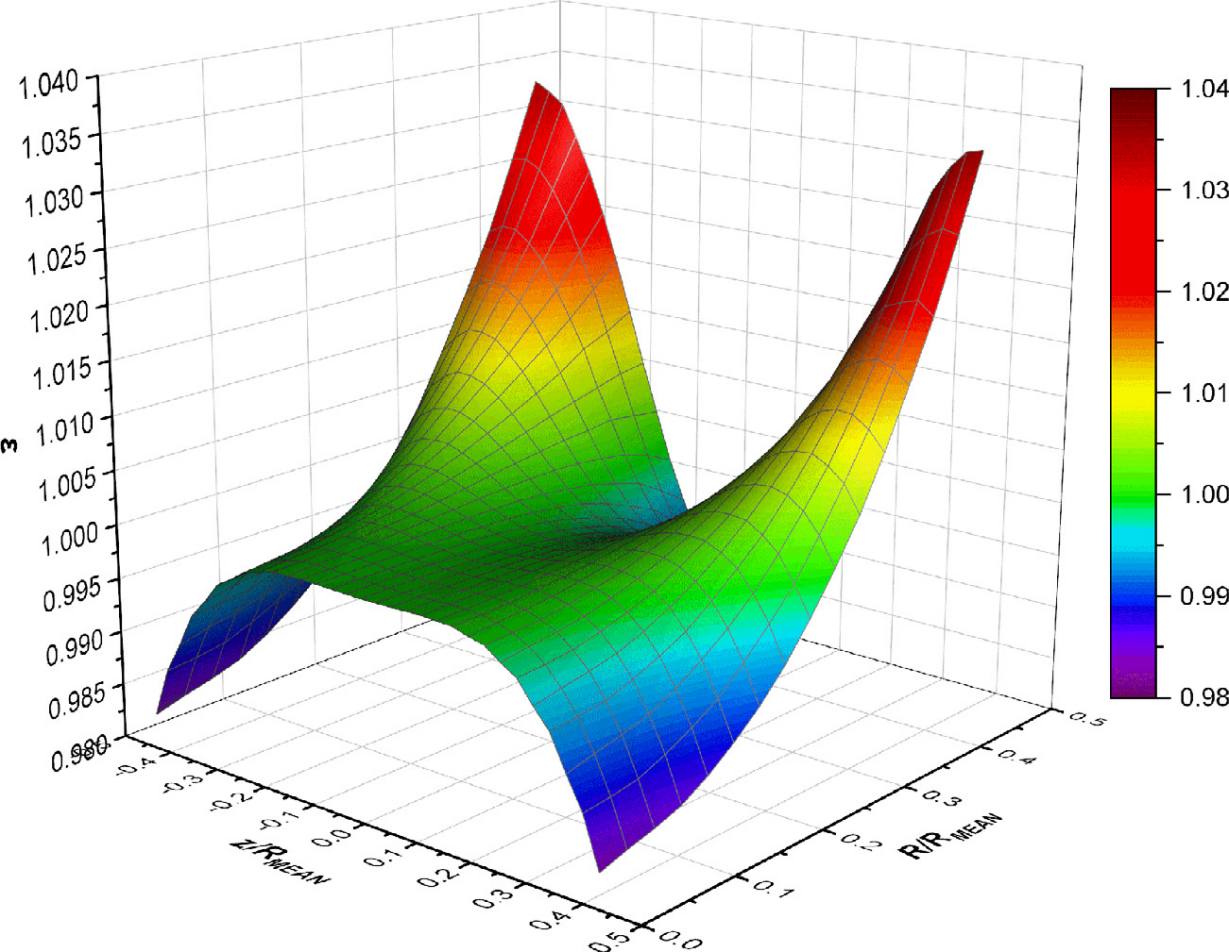
Normalised simulated magnetic flux distribution of the magnetic coils.

The normalised magnetic flux distribution obtained from the simulations are illustrated in Fig. 10. The surface plot shows half of the magnetic flux distribution as the other half is identical due to symmetry of the magnetic coils, where *R/R_MEAN_* = 0 corresponds to the simulated profile in Fig. 9. As the radial distance (*R/R_MEAN_*) increases, it can be observed that the magnetic field strength increases at the two corners, while decreases along the mid-plane (*z/R_MEAN_* = 0). The increase in magnetic field strength at the corners is mainly attributed to being in closer proximity to the individual magnetic coil windings (Fig. 6a). According to the simulated results, it was determined that a uniform magnetic field region of 20 × 26 mm (horizontal × vertical) was able to be achieved from this magnetic coil configuration.

#### 7.3.2 Relationship between current and magnetic flux density

**Fig. 11:**
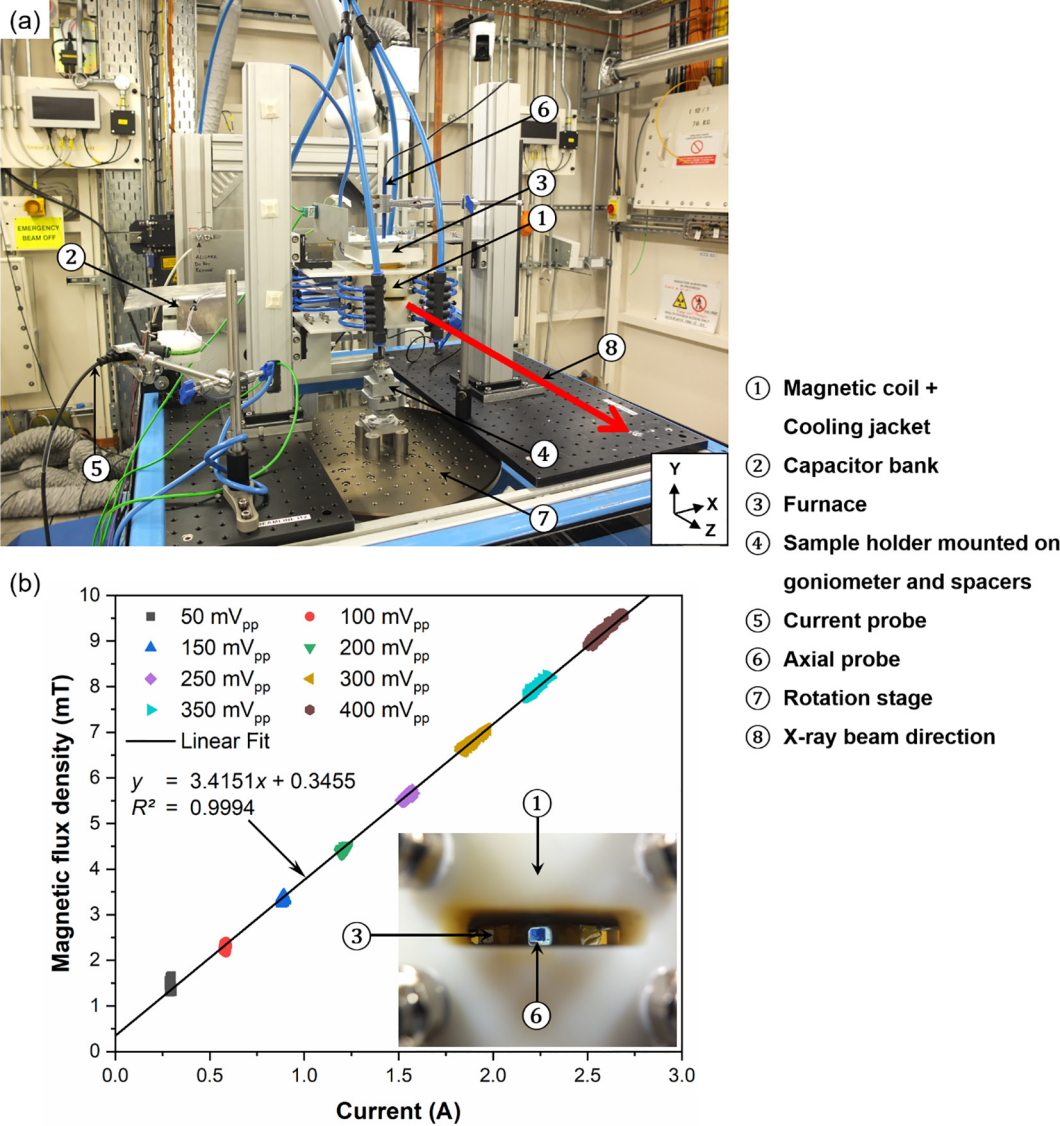
(a) The experimental setup of the apparatus, for carrying out the magnetic flux density measurements, as assembled on Sample Table 1 in Experimental Hutch 1 (EH1) of Beamline I12 - JEEP, Diamond Light Source, UK. (b) Linear relationship between measured current and magnetic flux density. The inset shows the position of the axial probe relative to the magnetic coil and furnace during measurements.

Compared to its DC counterpart, the achievable magnetic field strength in an AC system is typically much lower due to several factors, e.g., the circuit’s total impedance, frequency, impedance matching between the source (power supply) and the load (magnetic coils), the supply voltage, and Joule heating (*P* = *I^2^*Z). The correlation between the total current passing through the magnetic coil system and the generated magnetic flux density were investigated. Fig. 11a shows the experimental setup of the apparatus, for carrying out the magnetic flux density measurements, as assembled on Sample Table 1 in Experimental Hutch 1 (EH1) of Beamline I12. The magnetic flux density readings were measured by using an axial probe (Projekt Elektronik AS-NAP) which was connected to a gaussmeter (Projekt Elektronik Teslameter FM302). Note the measurements acquired were from the completed magnetic coil system, hence a different gaussmeter, capable of measuring high-frequency magnetic flux density measurements, was used. The axial probe was positioned in the centre of the magnetic coils and furnace (within the aperture) to mimic actual experimental conditions, where the sample is positioned (Inset of Fig. 11b). The gaussmeter and current probe were connected to the oscilloscope, which outputs the measurements in voltage. The highest sensitivity setting was selected for both the gaussmeter (2 V/20 mT) and current probe (100 mV/1 A). The measured outputs, shown in Fig. 11b, were recorded in real-time into Beamline I12′s data logging system and were acquired with a function generator input voltage at intervals of 50 mV_PP_ from 50–400 mV_PP_. The magnetic coils were also continuously cooled (one bar) with compressed air during the entire measurement period.

According to Fig. 11b, it was as expected that the magnetic flux density increased linearly with increasing current. Besides that, it could be observed that the magnetic flux appeared to drift from the scattering of data points at higher input voltages. This was attributed to the heating (*P* = *I^2^*Z) of the magnetic coils. As the magnetic coils heated up over time, the resistance of the magnetic coils increased, which in effect restricted the amount of current able to pass through the magnetic coils, as the input voltage was maintained constant.

### 7.4 Electromagnetic separation of inclusions in an aluminium alloy melt

The apparatus had successfully been used on a hypereutectic Al-20wt.%Si alloy. The results will be published elsewhere. The Al-20wt.%Si alloy was chosen because silicon has a similar density as that of a pure aluminium melt. The similar density of aluminium and silicon prevents the use of gravitational separation method. According to the equilibrium binary Al-Si phase diagram simulated by using the Thermo-Calc Software [33], there is a reasonable temperature range (~110°C) between the liquidus (~689°C) and eutectic (~577°C) temperature for the semi-solid processing. In the experiments, the alloy sample was first heated to above the liquidus temperature and held there to allow the melt temperature to fully homogenise. During cooling, primary silicon crystals (inclusions) nucleate and grow in the semi-solid region. The alternating magnetic field was applied just before the melt temperature crossed the liquidus temperature until the sample solidified (below eutectic temperature). The principle of electromagnetic separation relies on the difference in electrical conductivity between the bulk liquid metal and the inclusions inside. In this case, the aluminium melt at a temperature of ~700°C has a much higher conductivity (~3.8 × 10^6^ S/m), compared to the primary silicon crystals (~2 × 10^3^ S/m) [34], [35], [36]. Therefore, the aluminium melt experiences a much higher Lorentz force, which compresses the melt inwards. As a result, a pressure gradient is generated within the melt, forcing the non- or less-conductive inclusions to move away from the direction of the electromagnetic forces towards the wall of the crucible. The inclusions would finally be trapped at the vicinity of the wall due to the more intense Archimedes electromagnetic forces present [8], [10]. Concurrently, solidification of the sample was initiated from the outer surface and progressed inwards; this is known as progressive solidification [37]. Therefore, the non-metallic inclusions was completely immobilised at the wall and a “clean” central-region was produced once the sample had solidified.

**Fig. 12:**
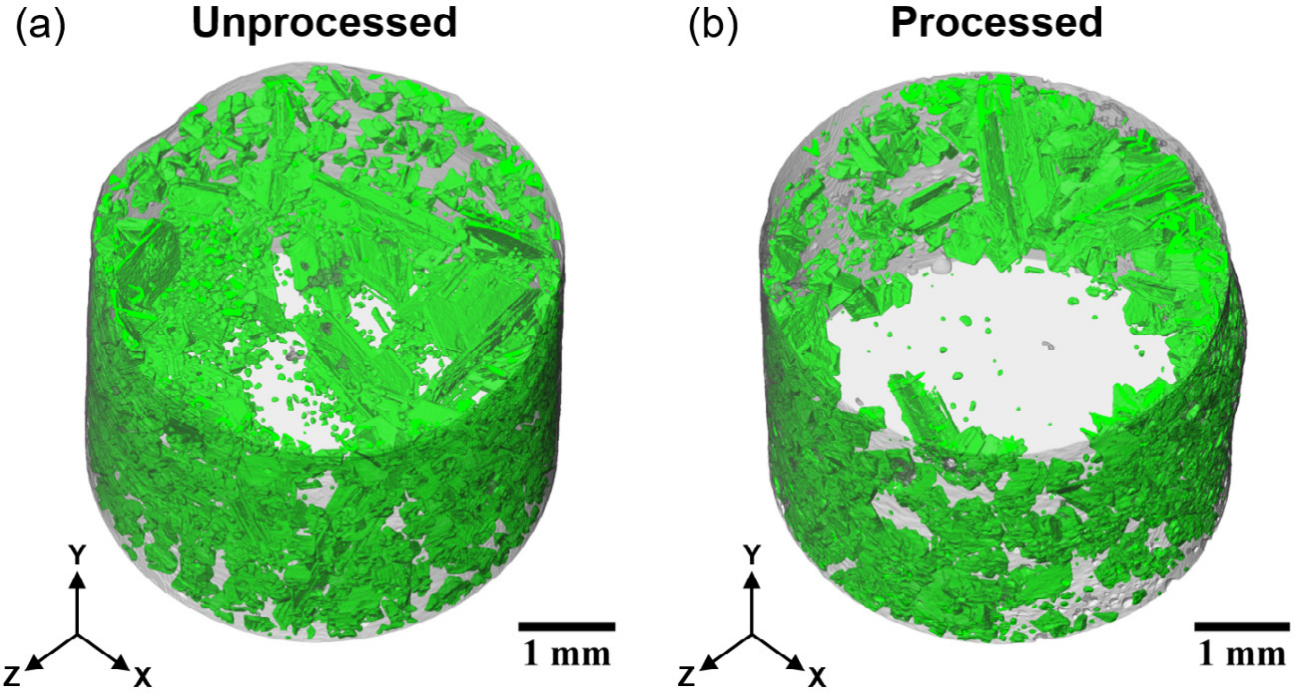
3D rendering of two Ø5 × 3.5 mm Al-20wt.%Si alloy tomography datasets. The aluminium matrix was rendered translucent to reveal the primary silicon crystals (green colour) within. (a) Unprocessed and (b) Processed.

Tomography scans were then performed on the solidified samples. A series of X-ray projections were acquired by rotating the sample via the rotation stage (No. 7 in Fig. 11a). The acquired X-ray projections were then reconstructed by using the Savu software [38] to obtain the stack of 2D cross-sectional images of the sample. This stack of 2D images were then imported into a dedicated visualisation software package, Avizo v2019.1 [39], to carry out the image segmentation to differentiate the various phases within the sample. After the individual phases within the sample were segmented, the 3D volume was then rendered as shown in Fig. 12a and Fig. 12b, for a section of the unprocessed and processed sample (Ø5 × 3.5 mm), respectively. The aluminium matrix was rendered translucent to allow for viewing of the primary silicon crystals (rendered green).

## 8 Hardware summary

In this manuscript, an electromagnetic field inclusion separation apparatus, consisting of a furnace and a magnetic coil system has been developed. A systematic and comprehensive characterisation of the hardware was performed through theoretical calculations, empirical measurements, and finite element simulations. The apparatus was commissioned at Beamline I12 - JEEP of Diamond Light Source and successfully used in two synchrotron X-ray imaging experiments (Proposal IDs: EE19256 & NT23362). Any interest in use of the system shall contact Prof. Jiawei Mi at the University of Hull, and to Dr. Thomas Connolley at Diamond Light Source. The summary of the capabilities and limitations of the hardware is listed in the following sub-sections.

### 8.1 Capabilities

The capabilities of the apparatus are summarised below:

• A maximum magnetic flux density of ~10 mT can be generated.
• Input voltage from the function generator can be controlled to generate different intensities of magnetic field.
• A large region of uniform magnetic field (±1%) of 20 × 26 mm (horizontal × vertical).
• Furnace capable of operating to temperatures up to ~850°C.
• Designed for conducting *in-situ* experiments (e.g., visible light, laser, X-ray beam, or neutron beam).

### 8.2 Limitations

The limitations of the apparatus are summarised below:

• Impedance mismatch resulted in reduced power transfer from the amplifier into the magnetic coils.
• Limited operation time of approximately three (3) minutes at a function generator input voltage of 400 mV_PP_, due to overheating of amplifier caused by reflected signal due to the mismatch.
• Frequency variation was not possible due to operation at resonance; capacitor bank with a different capacitance value would be required for a different frequency.
• Limited viewing window of 7 mm in height, due to optimal separation distance for achieving a uniform magnetic field.
• Limited viewing width of 8 mm, if furnace is placed within the magnetic coils.

## CRediT authorship contribution statement

**Billy Koe:** Conceptualization, Methodology, Validation, Formal analysis, Investigation, Data curation, Writing - original draft, Project administration. **Colin Abraham:** Conceptualization, Methodology, Validation, Resources, Writing - review & editing. **Chris Bailey:** Conceptualization, Methodology, Validation, Resources, Writing - review & editing. **Bob Greening:** Conceptualization, Methodology, Resources. **Martin Small:** Conceptualization, Methodology, Resources. **Thomas Connolley:** Conceptualization, Methodology, Writing - review & editing, Supervision, Funding acquisition. **Jiawei Mi:** Conceptualization, Methodology, Writing - review & editing, Supervision, Funding acquisition.

## Declaration of Competing Interest

The authors declare that they have no known competing financial interests or personal relationships that could have appeared to influence the work reported in this paper.
